# Membrane Proteins and Membrane Curvature: Mutual Interactions and a Perspective on Disease Treatments

**DOI:** 10.3390/biom13121772

**Published:** 2023-12-11

**Authors:** Peng Xie, Heng Zhang, Yatong Qin, Hehe Xiong, Changrong Shi, Zijian Zhou

**Affiliations:** State Key Laboratory of Vaccines for Infectious Diseases, Xiang An Biomedicine Laboratory & Center for Molecular Imaging and Translational Medicine, School of Public Health, Shenzhen Research Institute of Xiamen University, Xiamen University, Xiamen 361102, China; 32620211150851@stu.xmu.edu.cn (P.X.); 32620211150865@stu.xmu.edu.cn (H.Z.); 32620221150859@stu.xmu.edu.cn (Y.Q.); 32620210156084@stu.xmu.edu.cn (H.X.); changrongshi@xmu.edu.cn (C.S.)

**Keywords:** membrane protein, membrane curvature, interaction, disease treatment

## Abstract

The pathogenesis of various diseases often involves an intricate interplay between membrane proteins and membrane curvature. Understanding the underlying mechanisms of this interaction could offer novel perspectives on disease treatment. In this review, we provide an introduction to membrane curvature and its association with membrane proteins. Furthermore, we delve into the impact and potential implications of this interaction in the context of disease treatment. Lastly, we discuss the prospects and challenges associated with harnessing these interactions for effective disease management, aiming to provide fresh insights into therapeutic strategies.

## 1. Introduction

In biological systems, membranes are essential matrices of the cells, acting as selective barriers and gatekeepers for various cellular activities. For example, the plasma membrane separates individual cells from the complex external environment, facilitating the processes of cellular uptake, excretion, and intercellular message interaction. Eukaryotic cells contain many organelles that consist mainly of subcellular membranes, dividing the cytoplasm into multiple compartments and allowing for various biological processes to be carried out simultaneously and efficiently in each organelle [1,2]. Each membrane-bounded organelle has its characteristic shapes. Some organelles show relatively simple membrane shapes, such as lysosomes and peroxisomes, which are typically spherical. However, most organelles have complex membrane architectures. For example, the membrane structure of a mitochondrion forms a tubular network. Endoplasmic reticulum (ER) is mainly composed of cross-linked sheet and tubular membrane systems. The membrane structure of the Golgi apparatus is even more complex, with multiple shapes including flat cisternae, fenestrations, tubules, and vesicles. In addition to flat regions, the plasma membrane has invaginations and protrusions of different sizes and shapes. The characteristic shapes of these biological membranes are due to different local membrane curvatures. For example, in ER, a high cross-sectional curvature leads to tubule formation, while a low curvature promotes the formation of peripheral ER sheets [3,4].

Membrane curvature is not a passive geometric feature of biological membranes but rather interacts with membrane proteins as a highly adjustable property. On the one hand, membrane proteins are capable of generating membrane curvature [1,4]. On the other hand, the function and distribution of peripheral membrane proteins, integral membrane proteins, and pore-forming proteins within cells are affected by membrane curvature. Liposomes are suitable model membranes for investigating the effect of membrane curvature on membrane proteins. By controlling the size of liposomes, their membrane curvature can be altered, with smaller liposomes exhibiting a higher membrane curvature [2]. Using liposomes as model membranes to simulate different membrane curvatures, it was found that the major synaptic phosphoinositide phosphatase, synaptojanin 1 (Synj1), preferentially hydrolyzes PI(4,5)P_2_ in curved membranes to PI4P compared to membranes with a lower membrane curvature [5]. Most peripheral membrane proteins have membrane curvature sensors such as Bin/Amphiphysin/Rvs (BAR) domains and amphipathic lipid packing sensor (ALPS) motifs. Therefore, these peripheral membrane proteins are sensitive to membrane curvature, and the ability to sense different membrane curvatures endows proteins with a distinct spatial distribution in cells. For example, Nup133 and GMAP-210, which have ALPS motifs, are enriched on the nuclear envelope and cis-Golgi which have a high curvature [6,7]. For integral membrane proteins, when the membrane curvature is high, the integral membrane proteins in these membrane structures are physically stressed due to the bending of the lipid bilayer, which has a considerable impact on the structure and function of proteins. For example, a high membrane curvature significantly affects the activation gate conformation of the prototypical potassium channel KcsA [8]. Likewise, membrane curvature affects the distribution of integral membrane proteins embedded in the membrane, such as nicotinic acetylcholine and glutamate receptors. The Ca^2+^ and K^+^ ion channels are also concentrated in high-curvature regions such as post-synaptic regions [9,10,11,12]. In addition, membrane curvature also affects the membrane-binding ability and permeabilization of pore-forming proteins, resulting in different distributions of proteins on the membrane. For example, the hepatitis B virus X protein (HBx) functions as a pore-forming protein during the hepatitis B virus (HBV) life cycle, in which HBx specifically targets cardiolipin (CL) and induces membrane permeabilization based on CL concentration. More interestingly, phosphatidylethanolamine (PE) enhances HBx-induced membrane permeability by generating a negative curvature [13].

The interaction of membrane curvature with membrane proteins is disease-related. Firstly, the interaction between membrane curvature and membrane proteins affects the occurrence and the progression of multiple diseases. For example, enveloped viruses require membrane fusion to release the viral core into the cytoplasm before infecting cells. Viral proteins play a crucial role in the changing of curvatures during the membrane fusion [14]. Secondly, maintaining a typical membrane structure within cells requires the membrane curvature to interact with membrane proteins, such as the ER [15] and the T-tubule system in muscle cells [16]. The imbalance in the interaction between membrane curvature and membrane proteins will lead to a dysfunction and disease occurrence.

In this review, we first introduce the concept of membrane curvature and discuss how membrane proteins generate membrane curvature. Then, the effect of membrane curvature on the function and distribution of different kinds of membrane proteins is introduced. In addition, the impact of the interaction of membrane proteins with membrane curvature and a perspective on disease management are elaborated. Finally, we discuss the prospects and challenges of disease treatment through the interaction of membrane curvature with membrane proteins.

## 2. Membrane Curvature

**Figure 1:**
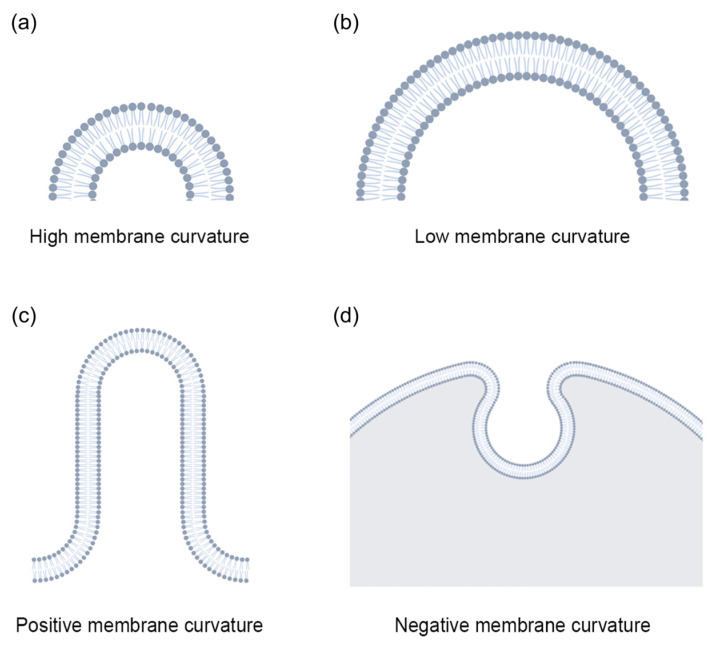
Schematic representation of different membrane curvatures. (**a**) High membrane curvature. (**b**) Low membrane curvature. (**c**) Positive membrane curvature. (**d**) Negative membrane curvature.

Membrane curvature is an important physical parameter that defines the morphology of cells, organelles, and local membrane structures [17]. For example, the shape of a point on the membrane surface can be described using two principal curvatures, c_1_ and c_2_, which are the reciprocals of the radii of two perpendicular arcs, R_1_ and R_2_, lying on the membrane plane (c_1_ = 1/R_1_ and c_2_ = 1/R_2_). If the radius is much larger than the membrane thickness d, such as R_1_ ≫ d, it is considered a low membrane curvature. When the radius is comparable to or only slightly larger than the membrane thickness, such as R_1_ ≥ d, it is considered a high membrane curvature. In addition, these principal curvatures can be used to describe the total curvature (J = c_1_ + c_2_) and the Gaussian curvature (K = c_1_c_2_) [4]. Specifically, we can describe microvilli, which are obvious protrusions, with a high membrane curvature (Figure 1a), and relatively flat regions on the plasma membrane with a low membrane curvature [4] (Figure 1b). Additionally, we can also describe the morphology of membrane structures using positive/negative membrane curvatures. A positive membrane curvature indicates regions of the membrane bending towards the cytoplasm, such as the early stages of vesicle budding, which exhibit a positive curvature (Figure 1c). On the other hand, a negative membrane curvature is the opposite, such as the invagination of the plasma membrane, which exhibits a negative membrane curvature [1] (Figure 1d).

## 3. Membrane Proteins Generate Membrane Curvature

Peripheral membrane proteins, integral membrane proteins, and the cytoskeleton can directly generate membrane curvature. They are not mutually exclusive but work together to generate membrane curvature.

### 3.1. Peripheral Membrane Proteins

Peripheral membrane proteins are membrane proteins that transiently bind to the membrane. They bind to the membrane through a combination of hydrophobic, electrostatic, and other non-covalent interactions [18]. The binding of peripheral membrane proteins to the membrane is reversible and can be disrupted by adding polar reagents such as a solution with an elevated pH or high salt concentrations [19]. Peripheral membrane proteins play critical roles in various cellular functions, including signal transduction [20], the formation of transport intermediates [21], and the transfer of molecules across membranes [22]. Moreover, due to their involvement in metabolic pathways, peripheral membrane proteins hold promise as targets for the treatment of diseases such as cancer, tuberculosis, and parasitic infections [19].

Peripheral membrane proteins can generate membrane curvature through the scaffolding mechanism. This mechanism involves protein monomers and oligomers, all of which possess intrinsic curvature, inducing membrane bending to generate membrane curvature. These proteins and oligomers need to meet the following criteria to generate membrane curvature: (1) having a strong affinity for the lipid headgroups that make up the phospholipid bilayer, (2) having sufficient rigidity to counteract the tendency for lipid bilayer relaxation, and (3) the energy for membrane–protein attachment exceeding the membrane-bending energy [4,23].

**Figure 2:**
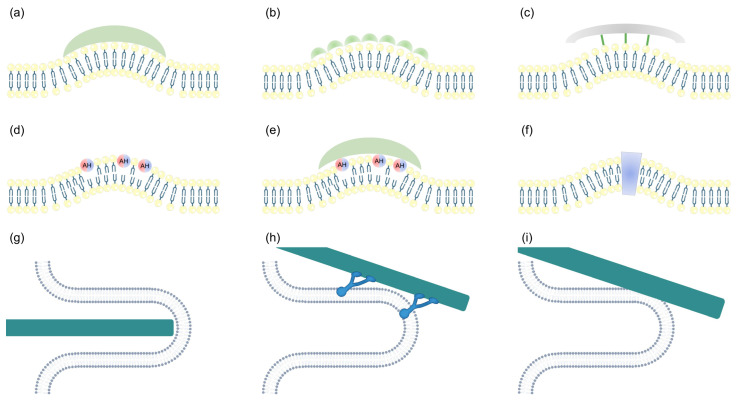
Schematic representation of membrane proteins generating membrane curvature. (**a**) The scaffolding mechanism by which peripheral membrane proteins with BAR domains can scaffold and bend the membrane by coordinating with the curvatures. Oligomers with intrinsic curvature are divided into direct (**b**) and indirect (**c**) scaffolding of lipid bilayers, depending on whether they bind to the membrane. (**d**) Hydrophobic insertion by which amphipathic helix (AH) insertion deforms the membrane by wedging effect. (**e**) Hydrophobic insertion and scaffolding work together to generate curvature. (**f**) Integral membrane proteins with intrinsic shapes, such as inverted conical shape, generate curvature by hydrophobic insertion. (**g**) Exerting mechanical force by the cytoskeleton which acts as an underlying scaffold to generate membrane curvature. (**h**) The cytoskeleton exerts pulling forces on the membrane through molecular motors. (**i**) The cytoskeleton exerts pulling forces on the tubular membrane.

Peripheral membrane proteins with BAR domains can meet the aforementioned conditions and, thus, generate membrane curvature (Figure 2a). Firstly, BAR domains have a curved surface and can bind to negatively charged membranes through electrostatic interactions [1]. Secondly, the bundled helices that make up the BAR domain provide sufficient rigidity to counteract the tendency of lipid bilayers to relax [23]. Finally, the formation of a small tube with a length of 5 nm and a radius of 11 nm only requires four BAR domains. At this point, the energy required for membrane curvature is approximately 28 *k_B_T*, while the electrostatic binding energy of four BAR domains is around 60 *k_B_T*, which is sufficient to generate membrane curvature [24,25]. Therefore, peripheral membrane proteins with BAR domains can force flat membranes to adopt the same curvature along the curved surface. Additionally, the ability of BAR domains to generate membrane curvature is limited. Although they can promote the formation of tubular membranes, they cannot generate the high membrane curvature required for membrane scission [26]. BAR domains are divided into four different structural categories: classical BAR domain, N-BAR domain with N-terminal AHs, F-BAR domain, and I-BAR domain. Both classical BAR domains and N-BAR domains have concave surfaces [27]. F-BAR domains also have a concave surface, but compared to N-BAR domains, F-BAR domains have a lower intrinsic curvature. Therefore, F-BAR domains generate a lower membrane curvature, and proteins containing F-BAR domains have higher affinity for membranes with a lower curvature [28,29]. In contrast to other BAR domains, I-BAR domains have a convex surface. Due to this structural difference, I-BAR domains generate opposite membrane curvatures compared to other BAR domains. In vitro, when using liposomes as model membranes, I-BAR domains induce an inward tubulation of liposomes, while endophilins containing BAR domains induce an outward tubulation of liposomes. In vivo, the overexpression of proteins containing I-BAR domains can lead to protrusions on the plasma membrane. Additionally, the insertion of AHs can promote membrane curvature for BAR domains with concave surfaces, but for I-BAR domains, the insertion of AHs inhibits membrane curvature generation [30,31].

Peripheral membrane protein monomers can oligomerize into rigid structures with intrinsic curvature, thereby generating membrane curvature through the scaffolding mechanism. For example, proteins with N-BAR or F-BAR domains can oligomerize on the membrane to form a protein coat, resulting in curvature and the formation of tubular membranes [26,28] (Figure 2b). Additionally, dynamin can generate membrane curvature by oligomerizing into long and rigid helical structures on the membrane [32]. During vesicle formation coated by clathrin, dynamin forms a helical structure around the neck of the clathrin-coated pit, generating membrane curvature and leading to the separation of the clathrin-coated pit from the plasma membrane [21]. Clathrin and coat protein complex I/II (COPI/II) can also form cage-like structures, forcing the membrane to adopt a spherical curvature [33,34]. However, clathrin and COPI/II cannot directly bind to the membrane; they require adaptor proteins to mediate their membrane binding (Figure 2c). By altering the adaptor proteins, the size of clathrin-coated vesicles can be changed [23].

Peripheral membrane proteins can also generate membrane curvature through hydrophobic insertion. They can insert hydrophobic domains (such as the hydrophobic surface of AHs) into the lipid bilayer, causing perturbations of lipid headgroups packing and generating local membrane curvature (known as the “wedging effect”) [4,35]. The hydrophobic insertion of peripheral membrane proteins is also influenced by membrane curvature. Due to the higher density of lipid packing defects on high-curvature membranes, peripheral membrane proteins preferentially insert into membranes with a higher curvature [36]. It is important to note that the depth of insertion of the hydrophobic domain into the lipid bilayer and the surface area occupied by the hydrophobic domain on the membrane both influence the generation of membrane curvature. To effectively generate membrane curvature, the hydrophobic domains of peripheral membrane proteins need to insert to a depth of approximately 1/3 to 1/2 of a single leaflet and cover approximately 10–25% of the membrane surface area [37,38].

The most common domain for hydrophobic insertion in peripheral membrane proteins is the AH (Figure 2d). For example, epsin1 lacks intrinsic curvature, and its N-terminal domain remains unfolded in solution. However, when the epsin N-terminal homology (ENTH) domain of epsin1 binds to the membrane, it can form an AH, which upon insertion generates membrane curvature, giving epsin1 the ability to generate membrane curvature [39]. Mutations in the hydrophobic region of the AH can abolish epsin1′s ability to generate membrane curvature [39]. Similarly, mutations in the AH of Arf1 and Sar1 also weaken their ability to generate curvature [40]. The hydrophobic insertion of Arf1 and Sar1 is influenced by membrane curvature, but the effect of membrane curvature on them is different. Arf1 has a weaker curvature-sensing ability [41], while Sar1, in addition to having a stronger curvature-sensing ability, also has a preference for binding to high-curvature membrane structures [42]. Sar1 has a strong ability to generate membrane curvature and can insert its AH into the membrane to generate the extreme curvature required for the fission of COPII vesicles [43]. α-synuclein, similar to epsin1, is intrinsically disordered, but when its N-terminal sequence binds to the membrane, it folds into an AH, thereby generating membrane curvature [44]. The hydrophobic insertion of AH is essential for cellular activities. For example, Pex11 plays a crucial role in peroxisome fission in vivo by inserting its AH into the lipid bilayer to generate a high membrane curvature [45]. Additionally, some peripheral membrane proteins generate membrane curvature by inserting hydrophobic loops into the lipid bilayer to act as wedges, such as EH-domain containing 2 (EHD2) [46,47].

Some peripheral membrane proteins generate membrane curvature through the combined action of scaffolding and hydrophobic insertion (Figure 2e). Peripheral membrane proteins such as endophilin and amphiphysin contain N-BAR domains, which can generate membrane curvature through the BAR domain and AH insertion. Mutations in the AH can weaken their ability to generate membrane curvature [26,48] but not completely abolish it, indicating that the scaffolding mechanism alone can still generate membrane curvature. Research has investigated the ability of proteins containing N-BAR domains to remodel membranes through the combined action of two mechanisms. It was found that vesicle formation is primarily attributed to the shallow insertion of the AH, while the synergy between the scaffolding and deep insertion of the AH leads to the formation of tubular membranes [49]. Additionally, pacsin can generate membrane curvature through the synergistic action of the F-BAR domain and hydrophobic loops, resulting in the formation of small tubes and small, uniform vesicles [27]. Human FCHo2 can generate membrane curvature through the F-BAR domain and N-terminal helix, and mutations in the N-terminal helix also weaken the ability of FCHo2 to generate membrane curvature [50].

### 3.2. Integral Membrane Proteins

In contrast to peripheral membrane proteins, integral membrane proteins are permanently embedded in the membrane and require the addition of detergents or non-polar solvents to extract them from the membrane. Integral membrane proteins can be further categorized into transmembrane proteins, which span both ends of the membrane, and integral monotopic proteins, which are attached to one side of the membrane but do not cross it. These proteins are essential for maintaining the membrane structure and carrying out various cellular processes. For example, lipid flippases are responsible for generating phospholipid asymmetry between the two leaflets of the membrane [51], mitochondrial carriers balance the export of newly synthesized ATP and the import of ADP in mitochondria [52], and anion-conductive PIEZO channels are involved in sensing touch [53].

Integral membrane proteins can also generate membrane curvature through hydrophobic insertion. Integral membrane proteins primarily generate membrane curvature through hydrophobic domains with intrinsic shapes [17], such as conical or inverted conical shapes (Figure 2f). For example, proteins with short hairpin transmembrane domains, such as reticulons, have their transmembrane domains occupying more space in the outer leaflet and inserting into the membrane in a shape similar to an inverted cone, resulting in membrane curvature [54]. A17, encoded by the poxvirus, is a transmembrane protein critical for the formation of crescent-shaped viral structures. A17 shares a similar topology with reticulons and is capable of generating high-membrane-curvature structures, such as vesicles and tubules with a diameter of 25 nm [55]. FAM134B is an ER-resident protein that regulates the size and shape of the ER. With the help of two wedge-shaped transmembrane hairpin domains, FAM134B can generate a high membrane curvature in the ER, leading to the formation of vesicles. These vesicles are subsequently engulfed by phagosomes, and the lack of FAM134B results in the expansion of ER sheets [15,56]. The insertion of the two AHs in FAM134B also promotes the formation of high-membrane-curvature structures. Mitochondrial ATP synthase forms V-shaped dimers in the membrane through the oligomerization of dimer-specific subunits *e* and *g* at an angle ranging from 70 to 90 degrees [57,58]. The V-shaped ATP synthase dimer generates local membrane curvature, which closely resembles the cristae of the inner membrane. Monomeric ATP synthase without the V-shaped conformation cannot generate membrane curvature [59]. Additionally, no other lipids or proteins are required to assist in the membrane remodeling process [59].

There are also some integral membrane proteins that can generate membrane curvature through the hydrophobic insertion of AHs and hydrophobic loops, without relying on hydrophobic domains with intrinsic shapes. The AH of peripherin-2/rds can insert into the lipid bilayer as a wedge to generate membrane curvature. In cellular experiments, it has been observed that peripherin-2/rds can induce the generation of the tubulovesicular membrane foci, providing evidence for its ability to generate membrane curvature [60]. The influenza virus transmembrane protein M2 can insert its AH into the lipid bilayer to generate membrane curvature, facilitating the budding of newly formed viral particles from the plasma membrane. The binding of cholesterol and the palmitoylation of the AH can both influence the interaction between AH and the membrane [61]. Synaptotagmin1 and Doc2b generate membrane curvature by using the hydrophobic loops at the C2 domain terminus as wedges in a Ca^2+^-dependent manner [62,63]. It is worth noting that a wide range of hydrophobic loop insertion is required to generate a high membrane curvature [64].

### 3.3. Mechanical Force by the Cytoskeleton

The cytoskeleton plays a crucial role in generating membrane curvature by exerting mechanical force on the membrane. Firstly, the cytoskeleton acts as an underlying scaffold, forming membrane curvatures at the macroscopic scale (with a radius of a few microns) [65] (Figure 2g). Additionally, the cytoskeleton can exert pulling forces on the membrane with the assistance of molecular motors (Figure 2h). Theoretical calculations suggest that the pulling force generated by ten molecular motors can meet the requirements for small tubules with a radius of 30 nm [23]. Furthermore, when the tubular membrane is attached to the cytoskeleton, the cytoskeleton can exert pulling forces on it (Figure 2i). This membrane remodeling, driven by the cytoskeleton, is essential for maintaining the morphology of organelles such as the ER, Golgi, and endosomes [66]. Moreover, the cytoskeleton is involved in remodeling the plasma membrane during cell division, the formation of membrane ruffles during phagocytosis, and the development of specialized cellular shapes [67].

## 4. Membrane Curvature Affects the Function and Distribution of Membrane Proteins

### 4.1. High Membrane Curvature to Peripheral Membrane Proteins

**Figure 3:**
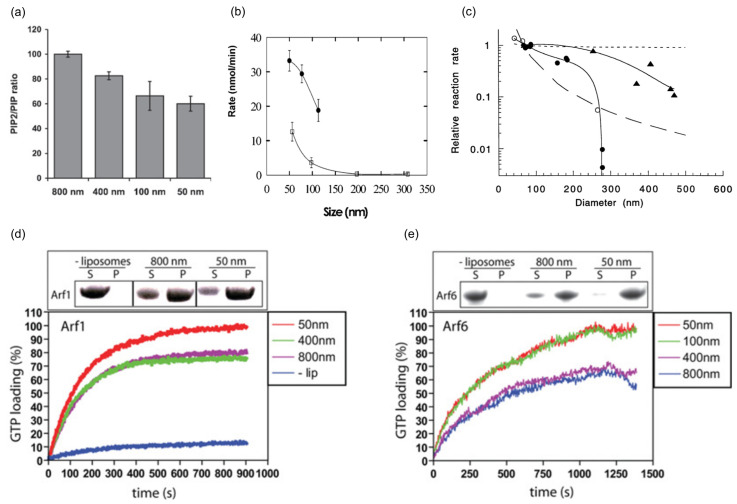
Effect of high membrane curvature on peripheral membrane protein function. (**a**) The ratio of PIP_2_/PIP for the different-sized liposomes. (**b**) Dependence of phosphatidylinositol-specific phospholipase C enzyme activity on vesicle size. The enzyme rate increases with a decrease in vesicle size. Vesicle composition was pure PI (Hollow box) or PI and PC (2/1 molar ratio) (Black dot). Reprinted with permission from ref. [72]. Copyright (2005) American Chemical Society. (**c**) Effect of vesicle size on PI 3-kinase (circles) and PtdIns 4-kinase (triangles) activity. Open circles and closed circles represent two independent experiments. Insets in (**d**,**e**) show liposome binding assays of Arf1 (**d**) and Arf6 (**e**) in the absence or presence of liposomes of different diameters. Reprinted with permission from ref. [74]. Copyright (2008) Portland Press, Ltd. (London, UK).

A high membrane curvature regulates the biological function of peripheral membrane proteins (Table 1). For example, ATG3 is a conjugating enzyme that operates on the surface of autophagosome membranes. During autophagosome growth, ATG3 catalyzes the covalent attachment of GABARAP-L1 (GL1) and its homologs to PE lipids on the membrane surface through a series of actions [68]. Comparing membranes with different curvatures, it has been found that the high-curvature structure favors the lipidation of GL1 [69]. Additionally, ATG3 possesses AHs that can sense membrane curvature. Therefore, the activity of ATG3 depends on AHs that can sense membrane curvature, and by regulating the hydrophobicity of AHs, lipidation can be inhibited or promoted [70]. In contrast to ATG3, which senses curvature through an AH, synj1 preferentially removes PI(4,5)P2, a molecule crucial for clathrin-mediated endocytosis, from membranes with a higher curvature using the BAR domain as a membrane curvature sensor [5] (Figure 3a). Alkaline phosphatase plays a crucial role in liver metabolism and bone development, and an increase in membrane curvature can enhance its activity [71]. Similar to alkaline phosphatase, the activity of phosphatidylinositol-specific phospholipase C enzyme on vesicles composed of phosphatidylinositol (PI) (with vesicle diameters in the range of 50–300 nm) also increases with membrane curvature [72] (Figure 3b). Moreover, when using liposomes containing phosphatidylcholine (PC) and PI as model membranes, the rate of phosphorylation of PI by phosphoinositide 3-kinase (PI 3-kinase) is strongly dependent on the curvature of the liposome (Figure 3c), with liposomes of approximately 50 nm in diameter phosphorylating much faster than liposomes with an average diameter greater than 300 nm [73]. Small G proteins belonging to the Arf family regulate numerous cellular processes by recruiting GTP, and studies have shown that the loading of GTP by Arf1 and Arf6 is dependent on membrane curvature [74] (Figure 3d,e). Arf1 interacts with the lipid membrane to drive the assembly of COPI coatings in a GTP-dependent manner, while ArfGAP 1 promotes the hydrolysis of GTP in Arf1, and its activity increases with membrane curvature, indicating that curvature not only affects GTP loading but also determines the timing and location of GTP hydrolysis [75,76]. Although a high membrane curvature can enhance the activity of many peripheral membrane proteins, the effect of membrane curvature on the activity of peripheral membrane proteins may not be uniform, even among homologs. Atg8-family proteins function through membrane tethering and fusion events during autophagosome formation. Human Atg8 orthologs, LC3B and GATE-16, exhibit different dependencies on membrane curvature for their membrane tethering reactions: LC3B is more efficient in driving tethering for small vesicles with a higher curvature, while GATE-16 is more active than LC3B for large vesicles with a lower curvature [77].

**Figure 4:**
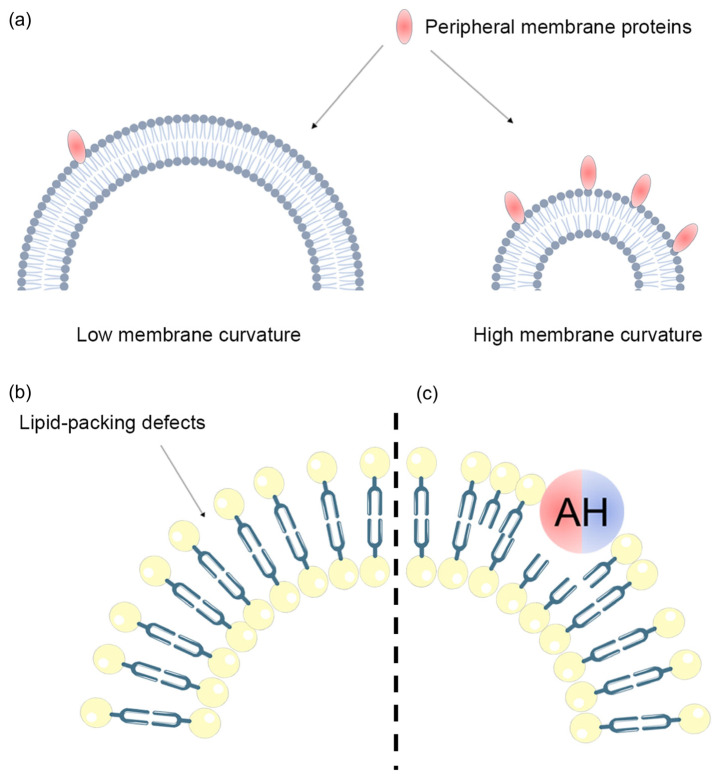
Effect of membrane curvature on peripheral membrane protein distribution. (**a**) Compared with membrane structures with low membrane curvature, peripheral membrane proteins have a higher binding ability to membranes with high membrane curvature. (**b**) High membrane curvature prevents lipids from filling the outer leaflet, resulting in lipid-packing defects. (**c**) If lipid-packing defects exist, proteins inserted into the membrane by hydrophobic action, such as those containing AH, can sense and stabilize membrane curvature.

Peripheral membrane proteins sense membrane curvature through various membrane curvature sensors and exhibit a higher binding affinity to membrane structures with a high curvature (Figure 4a). Peripheral membrane proteins like α-synuclein and annexin B12 preferentially bind to high-membrane-curvature structures through their AHs, and both the hydrophobic and hydrophilic faces of these helices influence their membrane binding [78]. Tumor protein D54 (TPD54), a member of the TPD52 family overexpressed in several cancer cells, contains four AHs (AH1-4), with AH3 displaying characteristics of an ALPS motif. Recent studies have shown that TPD54 predominantly binds to small cytosolic vesicles with a diameter of approximately 30 nm via AH2 and AH3, leading to its enrichment on these small vesicles [79]. Sec14-like 3 (Sec14L3), highly expressed in alveolar type II cells, utilizes its Sec14 domain as a lipid-packing sensor to recognize lipid-packing defects. Sec14L3 exhibits a stronger binding affinity to small liposomes with a diameter of 30 nm compared to giant liposomes with a diameter of 100 nm [80]. In addition, N-Ras with dual lipidation (palmitoyl and farnesyl) exhibits a stronger affinity for high-membrane-curvature structures [81]. The aforementioned peripheral membrane proteins primarily bind to the high-membrane-curvature structures through membrane curvature sensors that can insert hydrophobic residues. Therefore, the higher affinity of these peripheral membrane proteins for high-curvature membranes can be attributed to an increase in the density of lipid packing defects on high-curvature membranes (Figure 4b,c). Arfaptin2 senses curvature through its BAR domain, and active Arf1 loaded with GTP can recruit Arfaptin2 to model membranes that mimic the lipid composition of the Golgi apparatus. The recruitment of Arfaptin2 increases with membrane curvature, indicating that efficient recruitment requires a high membrane curvature [82]. Additionally, RNA-binding protein She2p, despite lacking known curvature-recognizing motifs, demonstrates a binding preference for model membranes with a high curvature [83]. COPII-coated vesicles play a crucial role in transporting cargo from the ER to the Golgi apparatus, and COPII exhibits a clear preference for high-curvature structures, although it can also bind to low-curvature-membrane structures [84].

### 4.2. High Membrane Curvature to Integral Membrane Proteins

A high membrane curvature significantly affects the function of integral membrane proteins embedded in the membrane (Table 2). Soluble N-ethylmaleimide-sensitive factor attachment protein receptors (SNAREs) play a crucial role in membrane fusion during the release of neurotransmitters and hormones from vesicles. Studies have shown that membrane curvature is a critical factor influencing fusion kinetics. Liposome-based experiments have demonstrated that the fusion rates between liposomes of different membrane curvatures vary, with liposomes of a high membrane curvature with a diameter of 40–50 nm exhibiting the fastest fusion rate [69]. Moreover, the number of SNARE complexes required to promote membrane fusion is not fixed and is greatly influenced by membrane curvature. For instance, a high-curvature liposome with a diameter of 40 nm requires only one synaptobrevin, while a low-curvature liposome with a diameter of 100 nm requires 23–30 synaptobrevins [85]. Mutations in synaptotagmin1 can promote fusion between SNARE-bearing liposomes with a high membrane curvature, but their activity is significantly reduced when a liposome curvature is low [63]. For human β2-adrenergic receptors (β2AR), reconstitution in liposomes of different curvatures revealed that a high membrane curvature affects β2AR oligomerization. Using fluorescence resonance energy transfer (FRET) between Cy3 and Cy5 labels on β2AR, researchers observed that some oligomers formed in membranes with a lower curvature dissociated in the presence of a high membrane curvature [86]. Similarly, experiments using liposomes with different membrane curvatures as model membranes have shown that a high membrane curvature inhibits the activation of Bax, a protein involved in apoptosis [87]. Diacylglycerol kinase (DGK) catalyzes PI cycling within cells to produce phosphatidic acid (PA). Systematically altering the curvature of the model membrane in which DGK is located revealed that DGK lacks acyl-chain specificity and exhibits low enzymatic activity in the absence of regions with a high membrane curvature. Moreover, the enzymatic activity of DGK is significantly enhanced in membrane structures with a high curvature, such as membrane fusion intermediates and membrane junctions [88].

**Figure 5:**
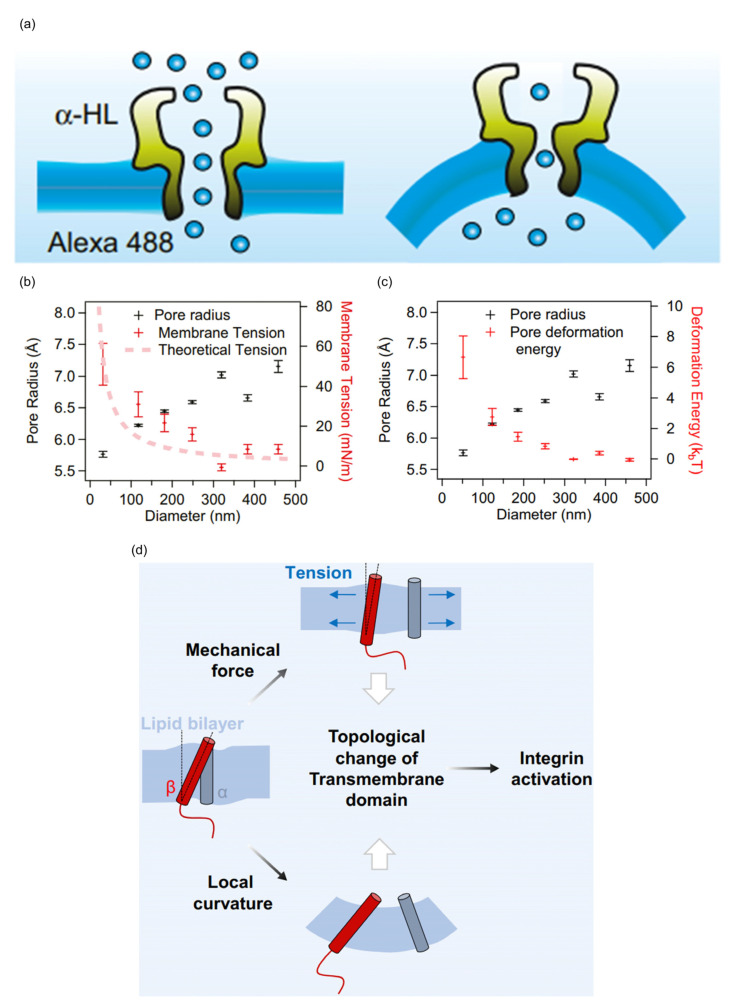
Effect of high membrane curvature on the structure and function of integral membrane proteins. (**a**) Schematic illustrations of high membrane curvature significantly changing the structure of the transmembrane protein α-HL. (**b**) Increasing membrane curvature reduces the pore radius (black crosses) of α-HL due to the increasing tension in the membrane. (**c**) Increasing membrane curvature reduces the pore radius (black crosses) of α-HL due to the increasing deformation energy in the membrane. Reprinted with permission from ref. [91]. Copyright (2014) Biophysical Society. (**d**) Schematic diagram of mechanical stimuli changing the topology of the integrin αIIbβ3 transmembrane domain, leading to activation of integrin αIIbβ3. The red cylinder represents the β3 transmembrane domain, and the gray cylinder represents the αIIb transmembrane domain. The interaction between them is weakened, resulting in the activation of integrin αIIbβ3.

Integral membrane proteins located in regions of a high membrane curvature experience physical stress due to the bending of lipid bilayers, which can affect their structure and consequently impact their function (Table 2). For instance, a single-channel optical analysis of the transmembrane β-barrel α-hemolysin (α-HL) on a 40 nm liposome demonstrated that a high membrane curvature exerts membrane tension and deformation energy on α-HL, resulting in the compression of the effective pore size and a reduction in the effective pore area [91] (Figure 5a–c). The tension-sensitive KcsA channel, which consists of an ion conduction pore and an activation gate, is also influenced by a high membrane curvature. A high membrane curvature can induce conformational changes in the activation gate, directly affecting the function of the ion channel [8]. The PIEZO channel, known for its sensitivity to forces, can sense changes in membrane curvature and respond to regulate corresponding physiological processes [93]. In addition, the patch-clamp technique was used to explore the impact of a PIEZO channel density on its pressure sensitivity or open probability in the absence of membrane tension. The results indicate that the PIEZO channel is essentially independent of density, and this property is crucial for the uniform transmission of force in cells [94]. Integrin αIIbβ3, a prototypic adhesion receptor, can be activated by mechanical stimuli, including an increase in membrane curvature. The increase in membrane curvature near integrin αIIbβ3 can modify the topology of its transmembrane domain, weakening transmembrane domain interactions and leading to integrin αIIbβ3 activation [95] (Figure 5d).

A high membrane curvature can lead to lipid-packing defects, which can facilitate the distribution of integral membrane proteins. In studying the distribution of the potassium channel KvAP in relation to membrane curvature, a model membrane was created using membrane nanotubes connected to unilamellar vesicles of the cell size. In addition, confocal microscopy was used to quantify the density and homogeneity of KvAP, while patch-clamp measurements were used to assess the activity of KvAP. The results indicate that as the membrane curvature of the nanotubes increases, the density of KvAP also increases [96]. Similarly, cytochrome b_5_ tends to be distributed in membrane regions with a higher curvature [97].

### 4.3. Positive/Negative Membrane Curvature to Pore-Forming Proteins

Pore-forming proteins are capable of creating unregulated pores in the target cell’s membrane, making them generally cytotoxic. These proteins can be classified into α pore-forming proteins and β-barrel pore-forming proteins based on their transmembrane channel structure. They play a role in specific cell death pathways, such as regulated cell death, which requires the activation and recruitment of pore-forming proteins [98]. Pore-forming proteins also regulate immune responses, as seen with gasdermin D, which forms pores to control the secretion of IL-1β by macrophages [99]. Furthermore, pore-forming proteins are crucial in disease infections. For example, during the plasmodium parasite infection of the liver, exported protein 2 secreted by sporozoites is a critical factor in the invasion of hepatocytes [100].

**Figure 6:**
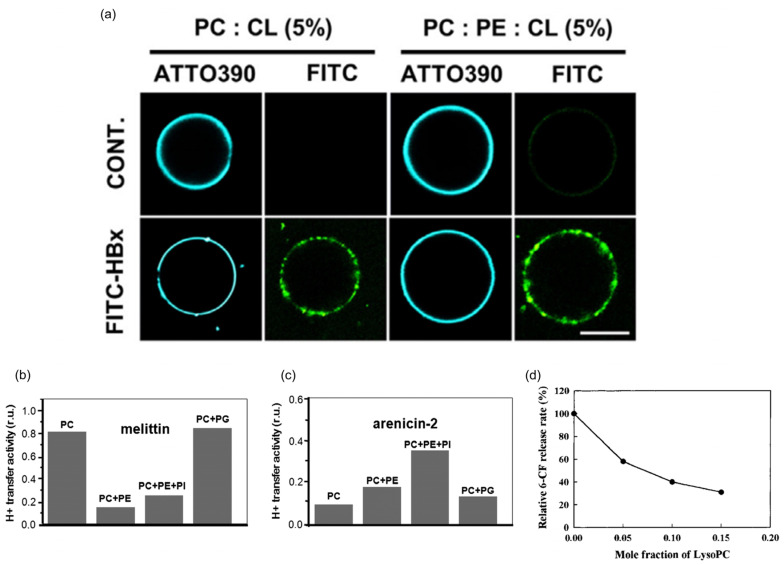
Effect of positive/negative membrane curvature on pore-forming protein function. (**a**) FITC-HBx targeting giant unilamellar vesicles containing PC:CL (95:5) or PC:PE:CL (65:30:5) was analyzed by confocal microscopy. The green color represents FITC-labeled HBx, and the blue color represents ATTO390-labeled vesicles. Scale bars indicate 10 μm. PTAs of the melittin (**b**) and arenicin-2 (**c**) in the membranes of different lipid compositions obtained by computer analysis of sigmoidal dependences. (**d**) Inhibitory effect of lyso-PC on aerolysin permeabilization. Reprinted with permission from ref. [104]. Copyright (2000) American Chemical Society.

Membrane curvature can indeed influence the function of pore-forming proteins (Table 3). For instance, during the HBV life cycle, the HBx protein, acting as a pore-forming protein, targets CL to generate mitochondrial membrane permeabilization. Interestingly, not only does the concentration of CL affect membrane permeabilization, but PE, which generates a negative curvature, can enhance HBx-induced membrane permeabilization. The study found that after labeling HBx with FITC, the targeting of HBx to the vesicles increased by incorporating PE into the vesicles, while maintaining the CL ratio in the vesicles unchanged [13] (Figure 6a). PE, which generates a negative spontaneous curvature, also affects the proton transfer activity (PTA) of cationic antimicrobial peptides. Experiments with PC-containing liposomes as model membranes have shown that replacing PC with PE leads to a decrease in the PTA of α-helical melittin and an increase in the PTA of β-structural arenicin-2 [101] (Figure 6b,c). Internal protein VI of adenovirus plays a role in adenovirus infection by binding to and lysing membranes. Interestingly, LPC, which generates a positive curvature, enhances its membrane-lysing ability [102]. LPC with a positive spontaneous curvature also enhances the pore-forming activity of colicin E1, while oleic acid, which generates a negative spontaneous curvature, reduces its activity [103]. However, the effect of membrane curvature on the aerolysin is opposite, as the permeabilization of the aerolysin is gradually inhibited with the increase in the mole fraction of LPC with a positive spontaneous curvature [104] (Figure 6d). Leukotoxins induce membrane bending, resulting in the formation of large pores that disrupt host cells. Leakage experiments with calcein-encapsulating liposomes have shown that liposomes composed of lipids with a negative spontaneous curvature exhibit enhanced leakage compared to liposomes composed of neutral-curvature lipids. Conversely, lipids with a positive spontaneous curvature completely eliminate leakage [105]. In summary, it is evident that the same membrane curvature can have different effects on the activity of pore-forming proteins, suggesting the need for further research to elucidate the underlying rules.

Moreover, a positive/negative membrane curvature can also impact the membrane-binding capacity of pore-forming proteins. For example, plantaricin A, a peptide pheromone with membrane-permeabilizing activity, is influenced by 1-palmitoyl-2-oleoyl-sn-glycerol-3-phosphoethanolamine, which generates a negative spontaneous membrane curvature, altering the binding mode of the peptide to the membrane [106].

## 5. Perspective on the Disease Treatments

### 5.1. Disease Progression

**Figure 7:**
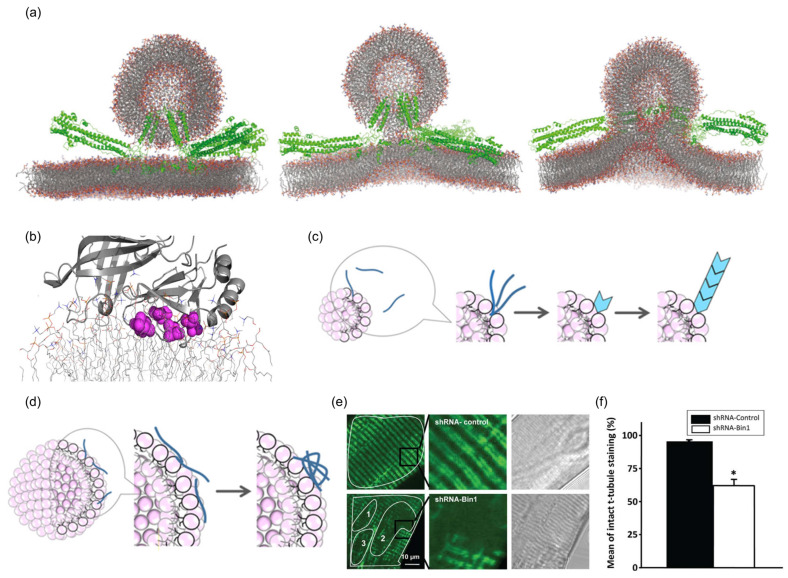
Effects of membrane protein interaction with membrane curvature on diseases. (**a**) Influenza hemagglutinin promotes acyl tail protrusion leading to stalk formation. (**b**) A model membrane depicting the viral protein 40 C-terminal domain penetrating deeply into the bilayer. (**c**) Schematic mechanism of the amyloid β peptide fibrillation in the presence of liposomes. In the presence of smaller liposomes, amyloid β peptide interacts weakly with the surface of the membrane, and this weak interaction promotes nucleation. (**d**) In the presence of larger liposomes, amyloid β peptide interacts with the membrane’s surface more strongly than smaller liposomes. This stronger interaction makes it difficult to induce nucleation and transforms some amyloid β peptides to amorphous aggregates. (**e**) Disrupted T-tubule structure in adult short-hairpin (sh) RNA targeting Bin1 (shRNA-Bin1) muscle. (left) Individually isolated flexor digitorum brevis muscle fiber that was electroporated with either shRNA-control or shRNA-Bin1 plasmid was stained with 100 nM DiOC5 that labeled intact t-tubule membrane structure. shRNA-Bin1 fibers exhibited loss or diffused of t-tubule staining in shRNA-Bin1 muscle fibers (n = 13), which, otherwise, were not observed in the shRNA-control fibers (n = 13). Enlarged images show that bifurcated t-tubule doublets characteristics are missing in the shRNA-Bin1 fibers (middle). Brightfield images (right) of cross-striated pattern in both shRNA-control and shRNA-Bin1 indicate healthy muscle fibers were chosen for DiOC5 staining. (**f**) Quantification of missing/diffused staining observed in shRNA-Bin1 when compared to shRNA-control fibers. Results are present as mean ± standard error of mean as tested for statistical significance by *t*-test. “*” represents *p* < 0.05.

The interaction between membrane curvature and membrane proteins plays a critical role in disease progression. For example, enveloped viruses like influenza virus require membrane fusion between the viral envelope and the cell membrane before infecting cells, and the membrane curvature changes continuously throughout the membrane fusion process. In the case of the influenza virus, viral proteins such as hemagglutinin can alter membrane curvature by increasing lipid tail protrusions and promoting stalk formation, widening, and bending to facilitate membrane fusion [14] (Figure 7a). After the influenza A virus infects cells, new virions are generated through reassembly on the plasma membrane. Many viral proteins, such as hemagglutinin and neuraminidase, generate changes in cell membrane curvature to promote virion assembly during budding from infected host cells [107]. Ebola viruses cause high fatality rates in human and non-human primates, with no proven treatment available. Viral protein 40 has the ability to generate membrane curvature by inserting hydrophobic residues deep into the lipid bilayer, thereby facilitating the formation and release of Ebola virus particles on the plasma membrane [108] (Figure 7b). Similarly, the non-structural protein 4A of dengue virus promotes the formation of host-cell-membrane-derived structures by inserting N-terminal α-helices into the membrane, providing a scaffold for viral replication [109]. The accessory human immunodeficiency virus-1 Nef protein plays a crucial role in promoting the internalization of T cell receptor molecules from the cell surface, which is essential for AIDS progression and viral replication. Nef’s function relies on its N-terminal myristoylation of the targeted membrane, and the rate of myristate insertion into the lipid bilayer depends on membrane curvature [110]. HBx, the smallest viral protein in HBV, is closely associated with chronic liver injury and cancer progression. HBx primarily localizes to mitochondria and induces mitochondrial membrane permeabilization by targeting CL, thereby altering membrane potential and dynamics. A negative curvature can enhance HBx-induced membrane permeabilization [13]. Alzheimer’s disease is characterized by the deposition of amyloid β peptides in the brain. The amyloid β peptides first fold from a disordered state to form the oligomeric nucleus and then extend to form amyloid fibrils. A high membrane curvature has been shown to promote β peptide nucleation, thereby accelerating the formation of amyloid fibrils (Figure 7c). Conversely, a lower membrane curvature leads to a decrease in fibril formation, shorter fibril length, and an increase in the number of amorphous aggregates [111,112] (Figure 7d). In summary, the interaction between membrane curvature and membrane proteins plays a crucial role in disease progression, influencing viral infection, cellular processes, and neurodegenerative disorders. Further research is needed to better understand these complex interactions and their potential therapeutic implications.

### 5.2. Disease Prevention

The interaction between membrane curvature and membrane proteins is crucial for maintaining a proper membrane structure within cells, thereby preventing diseases. The disruption of this interaction can potentially lead to the development of diseases. For example, the ER-resident protein FAM134B is responsible for generating a high membrane curvature in the ER to form vesicles. Deficiencies in FAM134B result in the expansion of ER sheets and diseases such as severe autonomic neuropathy type II [56,113]. Conversely, enhanced FAM134B function has been associated with the occurrence of vascular dementia and allergic rhinitis [15]. BIN1, which contains the N-BAR domain, is located on the tubular membrane invaginations (T-tubules) in skeletal myocytes. BIN1 is crucial for T-tubule formation due to the N-BAR domain’s ability to generate a high membrane curvature. The T-tubules play a critical role in maintaining synchronous calcium release, as both the Ca^2+^-releasing channel and the ryanodine receptor are located in the T-tubules. Knocking out BIN1 in skeletal muscle impairs T-tubule formation (Figure 7e,f) and disrupts intracellular calcium signaling [114]. Furthermore, mutations in the N-BAR domain of BIN1, such as R154Q, D151N, and K35N, have been found in patients with centronuclear myopathy. These mutations lead to T-tubule damage, with R154Q affecting membrane binding density and D151N inhibiting protein oligomerization during membrane binding [115]. The photoreceptor’s outer segments, which are crucial for vertebrate vision, consist of complex membranous structures. The precise stacking of hundreds of membranous discs, all with a high membrane curvature at their edges, is essential for the structure and function of the photoreceptor’s outer segments. This high membrane curvature is achieved through the insertion of AHs of peripherin-2/rds into the membrane leaflet, acting as wedges. Functional defects in peripherin-2/rds can lead to the disruption of organelle structure and retinal diseases [60]. In summary, the interaction between membrane curvature and membrane proteins is critical for disease prevention. Proper membrane curvature is essential for maintaining cellular structure and function, and disruptions in this interaction can contribute to the development of various diseases. Understanding the role of interaction in disease processes can provide insights for the development of therapeutic strategies.

## 6. Summary and Outlook

In this review, we first introduce membrane curvature. Then, we elucidate how membrane proteins generate membrane curvature and the effects of membrane curvature on the function and distribution of peripheral membrane proteins, integral membrane proteins, and pore-forming proteins, from which we can find that membrane proteins interact with membrane curvature in vivo. Finally, we discuss that the interaction of membrane proteins with membrane curvature can influence disease progression and generate and maintain typical membrane structure in vivo to prevent disease.

Looking ahead, further research is needed to deepen our understanding of how the interaction between membrane proteins and membrane curvature influences disease treatment, enabling us to develop viable therapeutic strategies. For example, recent studies have shown that the curvature-sensing properties of membrane proteins can enhance cancer immunotherapy by disrupting tumor-derived exosomes [116]. However, there is still much to learn about the interaction between membrane proteins and membrane curvature in the pathogenesis of various diseases, which limits our ability to leverage this interaction for therapeutic purposes. Therefore, future work should focus on elucidating the specific role of membrane curvature and membrane protein interactions in the pathogenesis of diseases, in order to provide insights for utilizing this interaction in the development of treatment strategies.

## Figures and Tables

**Table 1:** A summary of the effects of membrane curvature on peripheral membrane protein function.

Peripheral Membrane Proteins	Membrane Curvature	Effects	Reference
ATG3	High membrane curvature	Promotes lipidation of GL1.	[69]
Synaptojanin 1	High membrane curvature	Preferentially removes PI(4,5)P2.	[5]
LC3B (Human Atg8 orthologs)	High membrane curvature	Drives tethering more efficiently.	[77]
GATE-16 (Human Atg8 orthologs)	High membrane curvature	Reduces protein activity.	[77]
Alkaline phosphatase	High membrane curvature	Enhances enzyme activity.	[71]
Phosphatidylinositol-specific phospholipase C	High membrane curvature	The enzyme rate increases.	[72]
Phosphoinositide 3-kinase	High membrane curvature	The rate of phosphorylated PI increases.	[73]
ArfGAP 1	High membrane curvature	Promotes GTP hydrolysis in Arf1.	[75,76]

**Peripheral Membrane Proteins**

**Membrane Curvature**

**Effects**

**Reference**

**Table 2:** A summary of the effect of membrane curvature on the function and structure of integral membrane proteins.

Integral Membrane Proteins	Membrane Curvature	Effects	Reference
SNARE	High membrane curvature	Increases SNARE-mediated fusion rate.	[69]
	High membrane curvature	Reduces the number of SNARE complexes required for membrane fusion.	[85]
	Positive curvature	Inhibits SNARE-mediated membrane fusion by preventing the formation of membrane fusion intermediates with high negative curvature.	[89]
	Positive curvature	Inhibits the disassembly of SNARE complexes.	[90]
Synaptotagmin1 (mutant)	High membrane curvature	Promotes SNARE-mediated membrane fusion.	[63]
Human β_2_-adrenergic receptor	High membrane curvature	Inhibits β_2_-adrenergic receptor oligomerization.	[86]
Bax	High membrane curvature	Inhibits the activation of Bax.	[87]
Diacylglycerol kinase	Low membrane curvature	DGK lacks acyl-chain specificity and low enzyme activity.	[88]
α-hemolysin	High membrane curvature	Compression of the effective pore size of α-hemolysin and a reduction of about 40% of the effective pore area.	[91]
KcsA channel	High membrane curvature	The activation gate conformation changes.	[8]
Mechanosensitive ion channel	High membrane curvature	Induces activation of mechanosensitive ion channel function.	[92]

**Table 3:** A summary of the effects of membrane curvature on pore-forming protein function.

Pore-Forming Proteins	Membrane Curvature	Effects	Reference
Hepatitis B virus X protein	Negative curvature	Enhances HBx-induced membrane permeabilization.	[13]
α-helical melittin	Negative curvature	Reduces proton transfer activity.	[101]
β-structural arenicin-2	Negative curvature	Increases proton transfer activity.	[101]
Internal protein VI of adenovirus	Positive curvature	Enhances ability to lyse membranes.	[102]
Colicin E1	Positive curvature	Enhances the pore-forming activity of colicin E1.	[103]
	Negative curvature	Reduces the pore-forming activity of colicin E1.	[103]
Aerolysin	Positive curvature	Reduces the rate of pore formation.	[104]
Leukotoxin	Negative curvature	Enhances the pore-forming activity of leukotoxin.	[105]
	Positive curvature	Reduces the pore-forming activity of leukotoxin.	[105]

## Abbreviations

Abbreviation	Definition
AH	Amphipathic helix
ALPS motif	Amphipathic lipid packing sensor motif
BAR domain	Bin/Amphiphysin/Rvs domain
CL	Cardiolipin
COPI/II	Coat protein complex I/II
DGK	Diacylglycerol kinase
EHD2	EH-domain containing 2
ENTH domain	Epsin N-terminal homology domain
ER	Endoplasmic reticulum
FRET	Fluorescence resonance energy transfer
GL1	GABARAP-L1
HBV	Hepatitis B virus
HBx	Hepatitis B virus X protein
PA	Phosphatidic acid
PC	Phosphatidylcholine
PE	Phosphatidylethanolamine
PI	Phosphatidylinositol
PI 3-kinase	Phosphoinositide 3-kinase
PTA	Proton transfer activity
Sec14L3	Sec14-like 3
Sh	Short-hairpin
ShRNA-Bin1	Short-hairpin RNA targeting Bin1
SNARE	Soluble N-ethylmaleimide-sensitive factor attachment protein receptor
Synj1	Synaptojanin 1
TPD54	Tumor protein D54
α-HL	α-hemolysin
β2AR	β2-adrenergic receptors

## References

[1] McMahon H.T., Gallop J.L. (2005). Membrane curvature and mechanisms of dynamic cell membrane remodelling. Nature.

[2] Pomorski T.G., Nylander T., Cárdenas M. (2014). Model cell membranes: Discerning lipid and protein contributions in shaping the cell. Adv. Colloid. Interface Sci..

[3] Bigay J., Antonny B. (2012). Curvature, lipid packing, and electrostatics of membrane organelles: Defining cellular territories in determining specificity. Dev. Cell.

[4] Shibata Y., Hu J., Kozlov M.M., Rapoport T.A. (2009). Mechanisms shaping the membranes of cellular organelles. Annu. Rev. Cell Dev. Biol..

[5] Chang-Ileto B., Frere S.G., Chan R.B., Voronov S.V., Roux A., Di Paolo G. (2011). Synaptojanin 1-mediated PI(4,5)P2 hydrolysis is modulated by membrane curvature and facilitates membrane fission. Dev. Cell.

[6] Doucet C.M., Talamas J.A., Hetzer M.W. (2010). Cell cycle-dependent differences in nuclear pore complex assembly in metazoa. Cell.

[7] Cardenas J., Rivero S., Goud B., Bornens M., Rios R.M. (2009). Golgi localisation of GMAP210 requires two distinct cis-membrane binding mechanisms. BMC Biol..

[8] Ueki M., Iwamoto M. (2021). Fluorescent labeling in size-controlled liposomes reveals membrane curvature-induced structural changes in the KcsA potassium channel. FEBS Lett..

[9] Aimon S., Callan-Jones A., Berthaud A., Pinot M., Toombes G.E., Bassereau P. (2014). Membrane shape modulates transmembrane protein distribution. Dev. Cell.

[10] Fertuck H.C., Salpeter M.M. (1974). Localization of acetylcholine receptor by 125I-labeled alpha-bungarotoxin binding at mouse motor endplates. Proc. Natl. Acad. Sci. USA.

[11] MacKinnon R. (2003). Potassium channels. FEBS Lett..

[12] Unwin N. (2005). Refined structure of the nicotinic acetylcholine receptor at 4A resolution. J. Mol. Biol..

[13] You D.G., Cho Y.Y., Lee H.R., Lee J.H., Yu S.J., Yoon J.H., Yoo Y.D., Kim Y.J., Lee G.Y. (2019). Hepatitis B virus X protein induces size-selective membrane permeabilization through interaction with cardiolipin. Biochim. Biophys. Acta Biomembr..

[14] Pabis A., Rawle R.J., Kasson P.M. (2020). Influenza hemagglutinin drives viral entry via two sequential intramembrane mechanisms. Proc. Natl. Acad. Sci. USA.

[15] Bhaskara R.M., Grumati P., Garcia-Pardo J., Kalayil S., Covarrubias-Pinto A., Chen W., Kudryashev M., Dikic I., Hummer G. (2019). Curvature induction and membrane remodeling by FAM134B reticulon homology domain assist selective ER-phagy. Nat. Commun..

[16] Gowrisankaran S., Wang Z., Morgan D.G., Milosevic I., Mim C. (2020). Cells Control BIN1-Mediated Membrane Tubulation by Altering the Membrane Charge. J. Mol. Biol..

[17] McMahon H.T., Boucrot E. (2015). Membrane curvature at a glance. J. Cell Sci..

[18] Morozova D., Guigas G., Weiss M. (2011). Dynamic structure formation of peripheral membrane proteins. PLoS Comput. Biol..

[19] Boes D.M., Godoy-Hernandez A., McMillan D.G.G. (2021). Peripheral Membrane Proteins: Promising Therapeutic Targets across Domains of Life. Membranes.

[20] Monje-Galvan V., Klauda J.B. (2016). Peripheral membrane proteins: Tying the knot between experiment and computation. Biochim. Biophys. Acta.

[21] Kirchhausen T. (2000). Three ways to make a vesicle. Nat. Rev. Mol. Cell Biol..

[22] Stahelin R.V. (2008). Peripheral proteins as drug targets. Curr. Drug Targets.

[23] Zimmerberg J., Kozlov M.M. (2006). How proteins produce cellular membrane curvature. Nat. Rev. Mol. Cell Biol..

[24] Parthasarathy R., Groves J.T. (2006). Curvature and spatial organization in biological membranes. Soft Matter.

[25] Zimmerberg J., McLaughlin S. (2004). Membrane curvature: How BAR domains bend bilayers. Curr. Biol..

[26] Peter B.J., Kent H.M., Mills I.G., Vallis Y., Butler P.J., Evans P.R., McMahon H.T. (2004). BAR domains as sensors of membrane curvature: The amphiphysin BAR structure. Science.

[27] Goh S.L., Wang Q., Byrnes L.J., Sondermann H. (2012). Versatile membrane deformation potential of activated pacsin. PLoS ONE.

[28] Shimada A., Niwa H., Tsujita K., Suetsugu S., Nitta K., Hanawa-Suetsugu K., Akasaka R., Nishino Y., Toyama M., Chen L. (2007). Curved EFC/F-BAR-domain dimers are joined end to end into a filament for membrane invagination in endocytosis. Cell.

[29] Frost A., Perera R., Roux A., Spasov K., Destaing O., Egelman E.H., De Camilli P., Unger V.M. (2008). Structural basis of membrane invagination by F-BAR domains. Cell.

[30] Chen Z., Shi Z., Baumgart T. (2015). Regulation of membrane-shape transitions induced by I-BAR domains. Biophys. J..

[31] Saarikangas J., Zhao H., Pykäläinen A., Laurinmäki P., Mattila P.K., Kinnunen P.K., Butcher S.J., Lappalainen P. (2009). Molecular mechanisms of membrane deformation by I-BAR domain proteins. Curr. Biol..

[32] Pannuzzo M., McDargh Z.A., Deserno M. (2018). The role of scaffold reshaping and disassembly in dynamin driven membrane fission. Elife.

[33] Wenzel E.M., Schultz S.W., Schink K.O., Pedersen N.M., Nähse V., Carlson A., Brech A., Stenmark H., Raiborg C. (2018). Concerted ESCRT and clathrin recruitment waves define the timing and morphology of intraluminal vesicle formation. Nat. Commun..

[34] Chernomordik L.V., Kozlov M.M. (2003). Protein-lipid interplay in fusion and fission of biological membranes. Annu. Rev. Biochem..

[35] Has C., Das S.L. (2021). Recent developments in membrane curvature sensing and induction by proteins. Biochim. Biophys. Acta Gen. Subj..

[36] Hatzakis N.S., Bhatia V.K., Larsen J., Madsen K.L., Bolinger P.Y., Kunding A.H., Castillo J., Gether U., Hedegård P., Stamou D. (2009). How curved membranes recruit amphipathic helices and protein anchoring motifs. Nat. Chem. Biol..

[37] Campelo F., McMahon H.T., Kozlov M.M. (2008). The hydrophobic insertion mechanism of membrane curvature generation by proteins. Biophys. J..

[38] Li Z.L. (2018). Molecular dynamics simulations of membrane deformation induced by amphiphilic helices of Epsin, Sar1p, and Arf1. Chin. Phys. B.

[39] Ford M.G., Mills I.G., Peter B.J., Vallis Y., Praefcke G.J., Evans P.R., McMahon H.T. (2002). Curvature of clathrin-coated pits driven by epsin. Nature.

[40] Yorimitsu T., Sato K., Takeuchi M. (2014). Molecular mechanisms of Sar/Arf GTPases in vesicular trafficking in yeast and plants. Front. Plant Sci..

[41] Ambroggio E., Sorre B., Bassereau P., Goud B., Manneville J.B., Antonny B. (2010). ArfGAP1 generates an Arf1 gradient on continuous lipid membranes displaying flat and curved regions. EMBO J..

[42] Hanna M.G.t., Mela I., Wang L., Henderson R.M., Chapman E.R., Edwardson J.M., Audhya A. (2016). Sar1 GTPase Activity Is Regulated by Membrane Curvature. J. Biol. Chem..

[43] Lee M.C., Orci L., Hamamoto S., Futai E., Ravazzola M., Schekman R. (2005). Sar1p N-terminal helix initiates membrane curvature and completes the fission of a COPII vesicle. Cell.

[44] Westphal C.H., Chandra S.S. (2013). Monomeric synucleins generate membrane curvature. J. Biol. Chem..

[45] Opaliński Ł., Kiel J.A., Williams C., Veenhuis M., van der Klei I.J. (2011). Membrane curvature during peroxisome fission requires Pex11. EMBO J..

[46] Daumke O., Lundmark R., Vallis Y., Martens S., Butler P.J., McMahon H.T. (2007). Architectural and mechanistic insights into an EHD ATPase involved in membrane remodelling. Nature.

[47] Plomann M., Wittmann J.G., Rudolph M.G. (2010). A hinge in the distal end of the PACSIN 2 F-BAR domain may contribute to membrane-curvature sensing. J. Mol. Biol..

[48] Farsad K., Ringstad N., Takei K., Floyd S.R., Rose K., De Camilli P. (2001). Generation of high curvature membranes mediated by direct endophilin bilayer interactions. J. Cell Biol..

[49] Kahraman O., Langen R., Haselwandter C.A. (2018). Directed Supramolecular Organization of N-BAR Proteins through Regulation of H0 Membrane Immersion Depth. Sci. Rep..

[50] Henne W.M., Kent H.M., Ford M.G., Hegde B.G., Daumke O., Butler P.J., Mittal R., Langen R., Evans P.R., McMahon H.T. (2007). Structure and analysis of FCHo2 F-BAR domain: A dimerizing and membrane recruitment module that effects membrane curvature. Structure.

[51] Nintemann S.J., Palmgren M., López-Marqués R.L. (2019). Catch You on the Flip Side: A Critical Review of Flippase Mutant Phenotypes. Trends Plant Sci..

[52] Ruprecht J.J., Kunji E.R.S. (2021). Structural Mechanism of Transport of Mitochondrial Carriers. Annu. Rev. Biochem..

[53] Szczot M., Nickolls A.R., Lam R.M., Chesler A.T. (2021). The Form and Function of PIEZO2. Annu. Rev. Biochem..

[54] Zurek N., Sparks L., Voeltz G. (2011). Reticulon short hairpin transmembrane domains are used to shape ER tubules. Traffic.

[55] Erlandson K.J., Bisht H., Weisberg A.S., Hyun S.I., Hansen B.T., Fischer E.R., Hinshaw J.E., Moss B. (2016). Poxviruses Encode a Reticulon-Like Protein that Promotes Membrane Curvature. Cell Rep..

[56] Khaminets A., Heinrich T., Mari M., Grumati P., Huebner A.K., Akutsu M., Liebmann L., Stolz A., Nietzsche S., Koch N. (2015). Regulation of endoplasmic reticulum turnover by selective autophagy. Nature.

[57] Minauro-Sanmiguel F., Wilkens S., García J.J. (2005). Structure of dimeric mitochondrial ATP synthase: Novel F0 bridging features and the structural basis of mitochondrial cristae biogenesis. Proc. Natl. Acad. Sci. USA.

[58] Strauss M., Hofhaus G., Schröder R.R., Kühlbrandt W. (2008). Dimer ribbons of ATP synthase shape the inner mitochondrial membrane. EMBO J..

[59] Blum T.B., Hahn A., Meier T., Davies K.M., Kühlbrandt W. (2019). Dimers of mitochondrial ATP synthase induce membrane curvature and self-assemble into rows. Proc. Natl. Acad. Sci. USA.

[60] Khattree N., Ritter L.M., Goldberg A.F. (2013). Membrane curvature generation by a C-terminal amphipathic helix in peripherin-2/rds, a tetraspanin required for photoreceptor sensory cilium morphogenesis. J. Cell Sci..

[61] Thaa B., Levental I., Herrmann A., Veit M. (2011). Intrinsic membrane association of the cytoplasmic tail of influenza virus M2 protein and lateral membrane sorting regulated by cholesterol binding and palmitoylation. Biochem. J..

[62] Groffen A.J., Martens S., Díez Arazola R., Cornelisse L.N., Lozovaya N., de Jong A.P., Goriounova N.A., Habets R.L., Takai Y., Borst J.G. (2010). Doc2b is a high-affinity Ca^2+^ sensor for spontaneous neurotransmitter release. Science.

[63] Hui E., Johnson C.P., Yao J., Dunning F.M., Chapman E.R. (2009). Synaptotagmin-mediated bending of the target membrane is a critical step in Ca(2+)-regulated fusion. Cell.

[64] McMahon H.T., Kozlov M.M., Martens S. (2010). Membrane curvature in synaptic vesicle fusion and beyond. Cell.

[65] Doherty G.J., McMahon H.T. (2008). Mediation, modulation, and consequences of membrane-cytoskeleton interactions. Annu. Rev. Biophys..

[66] Leduc C., Campàs O., Joanny J.F., Prost J., Bassereau P. (2010). Mechanism of membrane nanotube formation by molecular motors. Biochim. Biophys. Acta.

[67] Rohn J.L., Baum B. (2010). Actin and cellular architecture at a glance. J. Cell Sci..

[68] Nguyen N., Shteyn V., Melia T.J. (2017). Sensing Membrane Curvature in Macroautophagy. J. Mol. Biol..

[69] Yang Y., Wu Z., Wang L., Zhou K., Xia K., Xiong Q., Liu L., Zhang Z., Chapman E.R., Xiong Y. (2021). Sorting sub-150-nm liposomes of distinct sizes by DNA-brick-assisted centrifugation. Nat. Chem..

[70] Nath S., Dancourt J., Shteyn V., Puente G., Fong W.M., Nag S., Bewersdorf J., Yamamoto A., Antonny B., Melia T.J. (2014). Lipidation of the LC3/GABARAP family of autophagy proteins relies on a membrane-curvature-sensing domain in Atg3. Nat. Cell Biol..

[71] Sesana S., Re F., Bulbarelli A., Salerno D., Cazzaniga E., Masserini M. (2008). Membrane features and activity of GPI-anchored enzymes: Alkaline phosphatase reconstituted in model membranes. Biochemistry.

[72] Ahyayauch H., Villar A.V., Alonso A., Goñi F.M. (2005). Modulation of PI-specific phospholipase C by membrane curvature and molecular order. Biochemistry.

[73] Hübner S., Couvillon A.D., Käs J.A., Bankaitis V.A., Vegners R., Carpenter C.L., Janmey P.A. (1998). Enhancement of phosphoinositide 3-kinase (PI 3-kinase) activity by membrane curvature and inositol-phospholipid-binding peptides. Eur. J. Biochem..

[74] Lundmark R., Doherty G.J., Vallis Y., Peter B.J., McMahon H.T. (2008). Arf family GTP loading is activated by, and generates, positive membrane curvature. Biochem. J..

[75] Bigay J., Casella J.F., Drin G., Mesmin B., Antonny B. (2005). ArfGAP1 responds to membrane curvature through the folding of a lipid packing sensor motif. EMBO J..

[76] Bigay J., Gounon P., Robineau S., Antonny B. (2003). Lipid packing sensed by ArfGAP1 couples COPI coat disassembly to membrane bilayer curvature. Nature.

[77] Taniguchi S., Toyoshima M., Takamatsu T., Mima J. (2020). Curvature-sensitive trans-assembly of human Atg8-family proteins in autophagy-related membrane tethering. Protein Sci..

[78] Jensen M.B., Bhatia V.K., Jao C.C., Rasmussen J.E., Pedersen S.L., Jensen K.J., Langen R., Stamou D. (2011). Membrane curvature sensing by amphipathic helices: A single liposome study using α-synuclein and annexin B12. J. Biol. Chem..

[79] Reynaud A., Magdeleine M., Patel A., Gay A.S., Debayle D., Abelanet S., Antonny B. (2022). Tumor protein D54 binds intracellular nanovesicles via an extended amphipathic region. J. Biol. Chem..

[80] Hishikawa D., Shindou H., Harayama T., Ogasawara R., Suwabe A., Shimizu T. (2013). Identification of Sec14-like 3 as a novel lipid-packing sensor in the lung. FASEB J..

[81] Larsen J.B., Kennard C., Pedersen S.L., Jensen K.J., Hatzakis N.S., Uline M.J., Stamou D. (2016). N-RAS Lipid Anchor Adsorption to Membranes as a Function of Lipid Composition and Curvature. Biophys. J..

[82] Ambroggio E.E., Sillibourne J., Antonny B., Manneville J.B., Goud B. (2013). Arf1 and membrane curvature cooperate to recruit Arfaptin2 to liposomes. PLoS ONE.

[83] Genz C., Fundakowski J., Hermesh O., Schmid M., Jansen R.P. (2013). Association of the yeast RNA-binding protein She2p with the tubular endoplasmic reticulum depends on membrane curvature. J. Biol. Chem..

[84] Bacia K., Futai E., Prinz S., Meister A., Daum S., Glatte D., Briggs J.A., Schekman R. (2011). Multibudded tubules formed by COPII on artificial liposomes. Sci. Rep..

[85] Hernandez J.M., Kreutzberger A.J., Kiessling V., Tamm L.K., Jahn R. (2014). Variable cooperativity in SNARE-mediated membrane fusion. Proc. Natl. Acad. Sci. USA.

[86] Mathiasen S., Tonnesen A., Christensen S.M., Fung J.J., Rasmussen S.G.F., Borrero E.E., Provasi D., Filizola M., Kobilka B.K., Stamou D.G. (2013). Membrane Curvature Regulates the Oligomerization of Human β2-Adrenergic Receptors. Biophys. J..

[87] Lucken-Ardjomande S., Montessuit S., Martinou J.C. (2008). Contributions to Bax insertion and oligomerization of lipids of the mitochondrial outer membrane. Cell Death Differ..

[88] Bozelli J.C., Jennings W., Black S., Hou Y.H., Lameire D., Chatha P., Kimura T., Berno B., Khondker A., Rheinstadter M.C. (2018). Membrane curvature allosterically regulates the phosphatidylinositol cycle, controlling its rate and acyl-chain composition of its lipid intermediates. J. Biol. Chem..

[89] James D.J., Khodthong C., Kowalchyk J.A., Martin T.F. (2008). Phosphatidylinositol 4,5-bisphosphate regulates SNARE-dependent membrane fusion. J. Cell Biol..

[90] Shin L., Wang S., Lee J.S., Flack A., Mao G., Jena B.P. (2012). Lysophosphatidylcholine inhibits membrane-associated SNARE complex disassembly. J. Cell Mol. Med..

[91] Tonnesen A., Christensen S.M., Tkach V., Stamou D. (2014). Geometrical membrane curvature as an allosteric regulator of membrane protein structure and function. Biophys. J..

[92] Foo A., Battle A.R., Marsh B.J., Hankamer B., Martinac B. (2010). Measuring the Release of Fluorescein from MscL-Loaded Liposomes with Stressed Lipid Bilayers. Biophys. J..

[93] Yang X., Lin C., Chen X., Li S., Li X., Xiao B. (2022). Structure deformation and curvature sensing of PIEZO1 in lipid membranes. Nature.

[94] Lewis A.H., Grandl J. (2021). Piezo1 ion channels inherently function as independent mechanotransducers. Elife.

[95] Kim J., Lee J., Jang J., Ye F., Hong S.J., Petrich B.G., Ulmer T.S., Kim C. (2020). Topological Adaptation of Transmembrane Domains to the Force-Modulated Lipid Bilayer Is a Basis of Sensing Mechanical Force. Curr. Biol..

[96] Aimon S., Toombes G., Yegor D., Bassereau P. (2011). Effects of Membrane Geometry on Voltage-Gated ion Channel Distribution Studied with a Model System. Biophys. J..

[97] Greenhut S.F., Bourgeois V.R., Roseman M.A. (1986). Distribution of cytochrome b5 between small and large unilamellar phospholipid vesicles. J. Biol. Chem..

[98] Vandenabeele P., Bultynck G., Savvides S.N. (2023). Pore-forming proteins as drivers of membrane permeabilization in cell death pathways. Nat. Rev. Mol. Cell Biol..

[99] Evavold C.L., Ruan J., Tan Y., Xia S., Wu H., Kagan J.C. (2018). The Pore-Forming Protein Gasdermin D Regulates Interleukin-1 Secretion from Living Macrophages. Immunity.

[100] Mello-Vieira J., Enguita F.J., de Koning-Ward T.F., Zuzarte-Luís V., Mota M.M. (2020). Plasmodium translocon component EXP2 facilitates hepatocyte invasion. Nat. Commun..

[101] Sychev S.V., Balandin S.V., Panteleev P.V., Barsukov L.I., Ovchinnikova T.V. (2015). Lipid-dependent pore formation by antimicrobial peptides arenicin-2 and melittin demonstrated by their proton transfer activity. J. Pept. Sci..

[102] Murayama T., Pujals S., Hirose H., Nakase I., Futaki S. (2016). Effect of amino acid substitution in the hydrophobic face of amphiphilic peptides on membrane curvature and perturbation: N-terminal helix derived from adenovirus internal protein VI as a model. Biopolymers.

[103] Sobko A.A., Kotova E.A., Antonenko Y.N., Zakharov S.D., Cramer W.A. (2004). Effect of lipids with different spontaneous curvature on the channel activity of colicin E1: Evidence in favor of a toroidal pore. FEBS Lett..

[104] Alonso A., Goñi F.M., Buckley J.T. (2000). Lipids favoring inverted phase enhance the ability of aerolysin to permeabilize liposome bilayers. Biochemistry.

[105] Brown A.C., Kieba I.R., Boesze-Battaglia K., Lally E.T. (2010). Aggregatibacter Actinomycetemcomitans Leukotoxin Disrupts Membranes By Inducing the Formation of An Inverted Hexagonal Lipid Phase. Biophys. J..

[106] Zhao H., Sood R., Jutila A., Bose S., Fimland G., Nissen-Meyer J., Kinnunen P.K. (2006). Interaction of the antimicrobial peptide pheromone Plantaricin A with model membranes: Implications for a novel mechanism of action. Biochim. Biophys. Acta.

[107] Chlanda P., Zimmerberg J. (2016). Protein-lipid interactions critical to replication of the influenza A virus. FEBS Lett..

[108] Soni S.P., Adu-Gyamfi E., Yong S.S., Jee C.S., Stahelin R.V. (2013). The Ebola virus matrix protein deeply penetrates the plasma membrane: An important step in viral egress. Biophys. J..

[109] Hung Y.F., Schwarten M., Hoffmann S., Willbold D., Sklan E.H., Koenig B. (2015). Amino Terminal Region of Dengue Virus NS4A Cytosolic Domain Binds to Highly Curved Liposomes. Viruses.

[110] Gerlach H., Laumann V., Martens S., Becker C.F., Goody R.S., Geyer M. (2010). HIV-1 Nef membrane association depends on charge, curvature, composition and sequence. Nat. Chem. Biol..

[111] Lomakin A., Chung D.S., Benedek G.B., Kirschner D.A., Teplow D.B. (1996). On the nucleation and growth of amyloid beta-protein fibrils: Detection of nuclei and quantitation of rate constants. Proc. Natl. Acad. Sci. USA.

[112] Terakawa M.S., Yagi H., Adachi M., Lee Y.H., Goto Y. (2015). Small liposomes accelerate the fibrillation of amyloid β (1-40). J. Biol. Chem..

[113] Kurth I., Pamminger T., Hennings J.C., Soehendra D., Huebner A.K., Rotthier A., Baets J., Senderek J., Topaloglu H., Farrell S.A. (2009). Mutations in FAM134B, encoding a newly identified Golgi protein, cause severe sensory and autonomic neuropathy. Nat. Genet..

[114] Tjondrokoesoemo A., Park K.H., Ferrante C., Komazaki S., Lesniak S., Brotto M., Ko J.K., Zhou J., Weisleder N., Ma J. (2011). Disrupted membrane structure and intracellular Ca^2+^ signaling in adult skeletal muscle with acute knockdown of Bin1. PLoS ONE.

[115] Wu T., Shi Z., Baumgart T. (2014). Mutations in BIN1 associated with centronuclear myopathy disrupt membrane remodeling by affecting protein density and oligomerization. PLoS ONE.

[116] Shin S., Ko H., Kim C.H., Yoon B.K., Son S., Lee J.A., Shin J.M., Lee J., Song S.H., Jackman J.A. (2023). Curvature-sensing peptide inhibits tumour-derived exosomes for enhanced cancer immunotherapy. Nat. Mater..

